# The Natural Product Curcumin as an Antibacterial Agent: Current Achievements and Problems

**DOI:** 10.3390/antiox11030459

**Published:** 2022-02-25

**Authors:** Chongshan Dai, Jiahao Lin, Hui Li, Zhangqi Shen, Yang Wang, Tony Velkov, Jianzhong Shen

**Affiliations:** 1 Department of Veterinary Pharmacology and Toxicology, College of Veterinary Medicine, China Agricultural University, No. 2 Yuanmingyuan West Road, Beijing 100193, China; jiahao_lin@cau.edu.cn (J.L.); szq@cau.edu.cn (Z.S.); wangyang@cau.edu.cn (Y.W.); 2 Beijing Key Laboratory of Detection Technology for Animal-Derived Food Safety, College of Veterinary Medicine, China Agricultural University, Beijing 100193, China; 3 Beijing Key Laboratory of Diagnostic and Traceability Technologies for Food Poisoning, Beijing Center for Disease Prevention and Control, Beijing 100193, China; lihui@bjcdc.org; 4 Department of Pharmacology & Therapeutics, School of Biomedical Sciences, Faculty of Medicine, Dentistry and Health Sciences, The University of Melbourne, Parkville, VIC 3010, Australia

**Keywords:** antibacterial resistance, curcumin, bacterial infection, molecular mechanism, nano-formulations

## Abstract

The rapid spread of antibiotic resistance and lack of effective drugs for treating infections caused by multi-drug resistant bacteria in animal and human medicine have forced us to find new antibacterial strategies. Natural products have served as powerful therapeutics against bacterial infection and are still an important source for the discovery of novel antibacterial drugs. Curcumin, an important constituent of turmeric, is considered safe for oral consumption to treat bacterial infections. Many studies showed that curcumin exhibited antibacterial activities against Gram-negative and Gram-positive bacteria. The antibacterial action of curcumin involves the disruption of the bacterial membrane, inhibition of the production of bacterial virulence factors and biofilm formation, and the induction of oxidative stress. These characteristics also contribute to explain how curcumin acts a broad-spectrum antibacterial adjuvant, which was evidenced by the markedly additive or synergistical effects with various types of conventional antibiotics or non-antibiotic compounds. In this review, we summarize the antibacterial properties, underlying molecular mechanism of curcumin, and discuss its combination use, nano-formulations, safety, and current challenges towards development as an antibacterial agent. We hope that this review provides valuable insight, stimulates broader discussions, and spurs further developments around this promising natural product.

## 1. Introduction

There is an urgent unmet medical need for new antibiotics for infections caused by multidrug-resistant (MDR) Gram-negative ‘superbugs’ *Pseudomonas aeruginosa*, *Acinetobacter baumannii,* and *Klebsiella pneumoniae* and Gram-positive methicillin-resistant *Staphylococcus aureus* (MRSA), vancomycin-resistant *S. aureus* (VRSA), and mobilized colistin resistance gene (MCR)-producing *Enterobacteriaceae* bacteria, which are resistant to almost all available antibacterial drugs [1]. The coronavirus disease 2019 (COVID-19) pandemic especially led to the increased clinical use of all antibiotics, which further promoted the development of bacterial resistance, highlighting the unmet medical need for new antibiotics [2].

Since the golden age of antibiotic discovery in the mid-20th century, natural products have served as the major foundation for the development of the majority of antibiotic drugs in clinical use to this very day [3]. Natural product antibiotics act by directly inhibiting the growth or killing the bacteria, acting as potentiators that augment or transform other agents or as immunomodulators to host cells or block pathogen virulence [1]. For example, a recent study from our group showed that two natural products, α-mangostin and isobavachalcone, could rapidly kill several types of MDR bacteria, including MRSA, VRSA, and MCR-producing Enterobacteriaceae bacteria in vitro [4].

Curcumin ((1E,6E)-1,7-bis-(4-hydroxy -3-methoxyphenyl)-hepta-1,6-diene -3,5-dione) is one of major active ingredients of turmeric extract, which is acquired from *Curcuma longa*, a type of herb belonging to the ginger family and widely growing in southern and south-western tropical Asia regions [5]. Curcumin is usually used as a coloring agent in foods or cooking. In China, curcumin has been approved as a food additive to improve animal’s productive performance [6]. Curcumin has been shown to possess direct broad spectrum antibacterial activities against Gram-negative and Gram-positive bacteria [6,7,8,9,10]. Curcumin also acts as an immunomodulator whereby it ameliorates bacterial infections by blocking the pathogen’s virulence factors and augmenting host-mediated immunity [11]. As a potential broad-spectrum antibacterial adjuvant to permeabilize the bacterial membrane, curcumin has a marked synergistic or additive anti-bacterial activity in combination with some traditional antibacterial drugs (e.g., polymyxin B, colistin, ciprofloxacin, and tetracycline) and natural active substances (e.g., berberine, and epigallocatechin gallate) [9,12]. Animal studies showed that topical curcumin was an effective treatment for localized trauma-caused skin infections [13]. Importantly, human trials showed that oral administration of curcumin was safe and effective for skin diseases, including psoriasis, infection, acne, skin inflammation, and skin cancer [14]. Bacterial infections also contribute to tumor formation, and curcumin combinations with some drugs have both anti-cancer and antibacterial activities that would provide a novel thyroid cancer treatment strategy [15]. In the present review, we survey the literature on the antibacterial properties and current underlying molecular mechanism of curcumin per se, curcumin-based combinations, and its nano-formulations, clinical trials, and major challenges, aiming to provide a prospective into the further clinical application of this promising antimicrobial candidate or broad-spectrum antibacterial adjuvant.

## 2. Chemical Structure and Biological Activity of Curcumin

**Figure 1:**
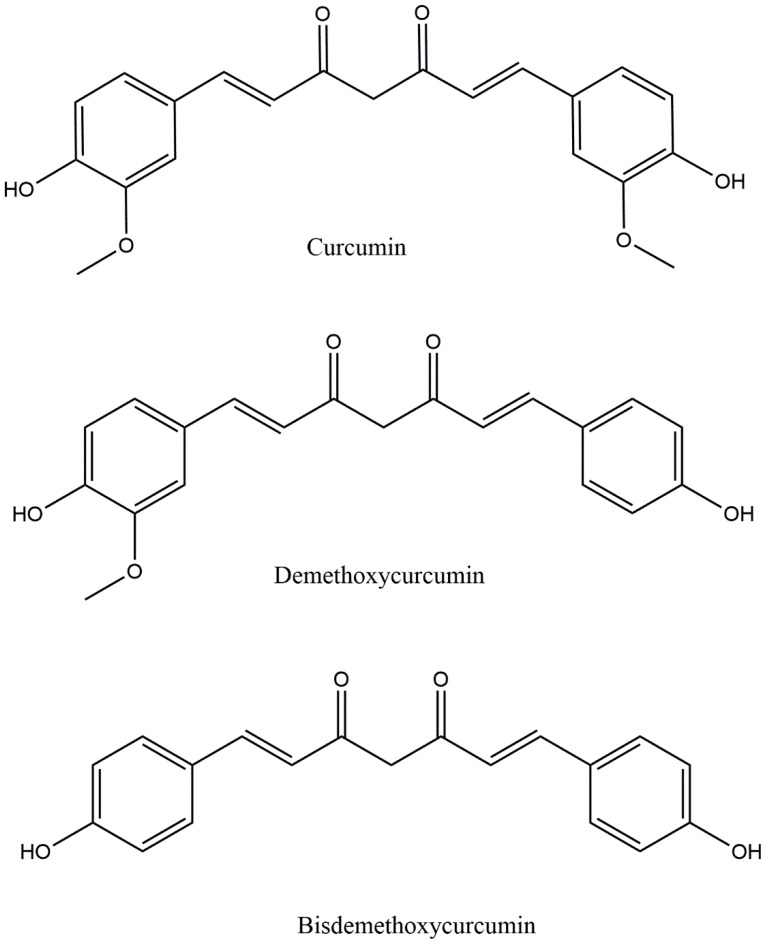
Chemical structures of curcuminoids.

**Figure 2:**
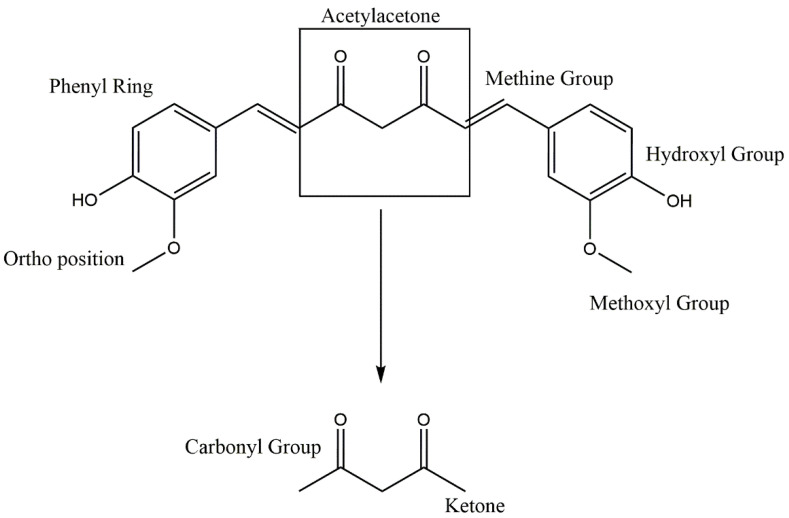
Functional groups in the molecular structure of curcumin.

The chemistry and structure of curcumin was first characterized in 1910 by Lampe and Milobedeska. Three years later, in 1913, they reported the synthesis of curcumin and confirmed its structure [16]. In 1953, Srinivasan reported the separation and quantification of components of curcumin using chromatography [17]. Curcumin is a mixture largely composed of three hydrophobic curcuminoids, namely, demethoxycurcumin (DMC), bisdemethoxycurcumin (BDMC), and curcumin, in the proportion of 17: 3: 77 (Figure 1) [18]. From a structural viewpoint, curcumin, DMC, and BDMC all contain two aromatic feruloyl rings with orthomethoxy phenolic OH groups (Figure 2). The highly polar aromatic rings are symmetrically connected via a seven-carbon aliphatic chain and two α, -unsaturated carbonyl groups (e.g., β-diketonemoiety) [19]. This seven-carbon aliphatic chain structure is responsible for the hydrophobic nature of curcumin, which makes it practically insoluble in water; however, solubility can be achieved in ethanol, dimethyl sulfoxide (DMSO), methanol, and acetone [19]. Curcumin displays a maximum ultraviolet (UV)-absorption (λmax) peak at 430 nm, which is due to the two feruloyl aromatic ring structure [19]. Curcumin has two molecular configurations, bis-keto and enolate. Its bis-keto form predominates under acidic, neutral, and solid phase conditions, whereas its enolic form is predominantly found under alkaline conditions [20]. 

Documented biological activities of curcumin include antimicrobial, antioxidant, anti-inflammatory, neuroprotective, anticancer, and immuno-modulatory activities [20]. Due to its various biological activities, curcumin has been used extensively in traditional medicine for the treatment of various illnesses including autoimmune, neurological, diabetic, cardiovascular, and infectious disease [5,21]. In the proceeding discussions, we elaborate on the antibacterial activities of curcumin, its mechanism of action, and barriers associated with its clinical application as an antibiotic therapy.

## 3. Antibacterial Activity of Curcumin

In 1949, Schraufstatter and colleagues were the first to report the antibacterial properties of curcumin [22]. In the past seventy years, there have been several studies of the broad-spectrum inhibitory effect that curcumin exhibits against various Gram-negative and Gram-positive bacteria, including *A. baumannii*, *E. faecalis*, *K. pneumoniae*, *P. aeruginosa*, *Bacillus subtilis* (*B. subtilis*), *Staphylococcus epidermidis*, *Bacillus cereus* (*B. cereus*), *Listeria innocua*, *Streptococcus pyogenes*, *S. aureus*, *Helicobacter pylori* (*H. pylori*), *Escherichia coli* (*E. coli*), *Salmonella enterica* serotype Typhimurium, and *Streptococcus mutans* (Details shown in Table 1) [6,8,10,23,24]. Importantly, curcumin also exhibits marked antibacterial activities against MDR-isolates, such as polymyxin-resistant *K. pneumoniae* and MRSA [9,10,24]. A recent study by Batista de Andrade Neto et al., reported that minimum inhibitory concentration (MIC) values for curcumin against clinical isolates of MRSA were in the range of 125–500 μg/mL [25]. Another study by Yasbolaghi Sharahi et al., reported that MICs of curcumin against MDR-*A. baumannii*, *P. aeruginosa* and *K. pneumoniae* were in the range of 128–512 μg/mL [8]. Notably, there were significant differences in the MICs of curcumin against certain stains reported by different research groups [26]. This may be due to the difference in solubility of curcumin in the different vehicles (e.g., water, DMSO, and ethanol) used by each research group [26]. In addition, these differences may be related to the MIC test methodology, impact of the vehicle against the bacterial outer membrane, and purity of the curcumin used in the study [27]. 

## 4. Mechanisms of the Antibacterial Action of Curcumin

### 4.1. Cell Membrane Disruption

Curcumin and its two analogs, DMC and BDMC, have been shown to possess antibacterial activity against a wide range of bacteria [23]. Studies have shown that curcumin can damage the permeability and integrity of bacterial cell membranes in both Gram-positive and -negative bacteria, finally leading to bacterial cell death [47]. Curcumin’s lipophilic structure allows it to directly insert into liposome bilayers, which in turn enhances the bilayer permeability [47]. Solid-state nuclear magnetic resonance (NMR) spectroscopy studies revealed that curcumin can insert deep into the membrane in a trans-bilayer orientation, resulting in disordering 1,2-dipalmitoyl-sn-glycero-3-phosphocholine (DPPC) membranes and influencing exocytotic and membrane fusion processes [48]. Tyagi et al., demonstrated that curcumin at a concentration of 100 μM can induce permeabilization of both *S. aureus* and *E. coli* cell walls [49]. This membrane permeabilization property could account for the direct bacterial killing effect of curcumin against Gram-positive and -negative bacteria [49]. Indeed, the increase in membrane permeabilization of bacteria caused by curcumin could increase the uptake of other drugs [50]. This is a critical mechanism to explain the synergistic effect of curcumin combination therapy with other antibiotic drugs or natural products, as discussed in detail below. 

### 4.2. Inhibition of Bacterial Quorum Sensing System and Biofilm Formation 

Quorum sensing (QS) system is a cell–cell communication system that is ubiquitously used in microbial communities to monitor their population density and adapt to external environment [51]. To date, there are three main QS systems, (1) the acylhomoserine lactone (AHL) QS system in Gram-negative bacteria; (2) the autoinducing peptide (AIP) QS system in Gram-positive bacteria, and (3) the autoinducer-2 (AI-2) QS system, which is in both Gram-negative and Gram-positive bacteria. It is well-known that QS systems play critical role in the formation and maturation of bacterial biofilms, which are associated with about 80% microbial infections [52]. Bacteria growing in biofilms are largely protected from antibiotics or host immune cells, leading to the failure of antimicrobial therapy [52]. QS systems are the master controllers for the entire process of biofilm formation, including bacterial adhesion, biofilm development, and maturation. Therefore, the discovery of new inhibitory compounds targeting bacterial QS systems is an important strategy to control bacterial biofilm formation and resistance. 

**Figure 3:**
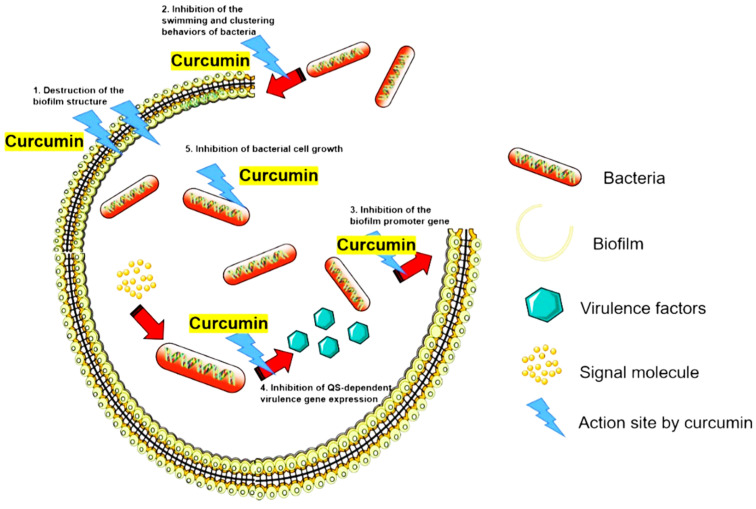
Inhibitory effect of curcumin against the bacterial quorum sensing (QS) system. The main mechanisms of curcumin in QS inhibition involve (1), destruction of the biofilm structure; (2) inhibition of bacterial swimming and clustering behavior; (3) inhibition of the expression of biofilm promotor genes; (4) inhibition of the gene expression of QS-dependent virulence; (5) inhibition of bacterial cell growth [35,36,38,50,53,54,55].

Several studies reported that curcumin inhibits bacterial QS systems/biofilm formation and prevents bacterial adhesion to host receptors in various species, including *S. aureus*, *E. faecalis*, *E. coli*, *Streptococcus mutans, Listeria monocytogenes*, *H. pylori*, *P. aeruginosa*, *Serratia marcescens*, *Aeromonas hydrophila* and *A. baumannii* [36,38,50,53,54,55]. We have summarized the QS system’s curcumin targets in various bacteria in Table 2. In addition, Figure 3 provides an overview of the inhibitory mechanisms of curcumin against biofilm formation, inhibition of bacterial swimming/clustering behaviors, and inhibition of virulence [35,36,38,50,53,54,55]. Interestingly, available data suggest that the autoxidation of curcumin could also contribute to the inhibition of biofilm formation [56]. For example, curcumin was shown promote the production of lactate dehydrogenase (LDH) in *P. aeruginosa, S. aureus,* and *E. faecalis,* wherein the curcumin/LDH complex exhibited antibacterial and anti-biofilm activities [56]. Clearly, the anti-biofilm properties of curcumin increase its potential as a tractable anti-infective agent. 

### 4.3. Inhibition of Cell Division 

**Figure 4:**
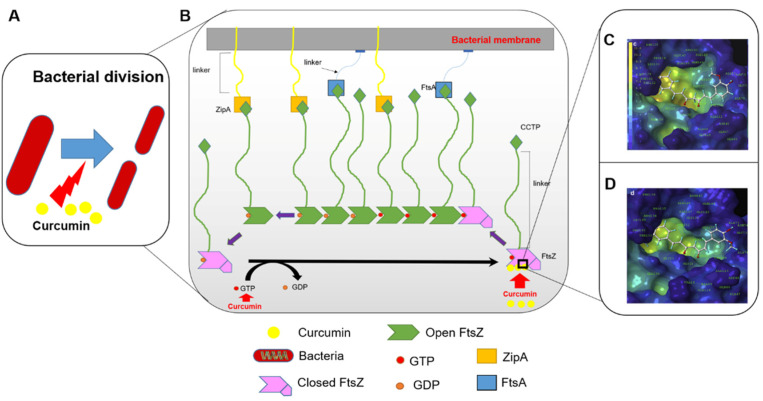
Curcumin inhibits the bacterial division by blocking FtsZ assembly. (**A**), working model of curcumin for the inhibition of bacterial division. (**B**), curcumin can activate the activity of GTP and interact with FtsZ, blocking the FtsZ assembly [65]. (**C**,**D**), the interaction site of FtsZ with curcumin in *E. coli* and *B. subtilis* strains, respectively [66].

Inhibition of bacterial cell division is an important mechanism of curcumin’s antibacterial activity [23,64]. Filament temperature-sensitive protein Z (FtsZ) is shown to be essential for bacterial cell division [64,65]. It consists of an N-terminal polymerization domain connected to a highly conserved C-terminal peptide (CCTP) of ~eight amino acids by an intrinsically disordered linker region of variable length (50 amino acids in *E. coli*). FtsZ associates in a GTP-dependent manner to form polymers [64]. This process is coupled to the conversion between closed and open conformations of FtsZ and plays a critical role in the formation of the Z ring of FtsZ. The polymerized FtsZ filaments attach to the cytoplasmic membrane through membrane anchors ZipA and FtsA, mediated by the CCTP of FtsZ (Figure 4). Rai et al., showed that curcumin blocks the formation of the cytokinetic Z ring through direct interaction with FtsZ in *B. subtilis* and *E.coli* [64]. In addition, curcumin also increased the GTPase activity of FtsZ, which in turn aborted the polymerization process [64]. Molecular docking of curcumin to the *E. coli* FtsZ structure suggests binding occurs within the GTPase catalytic pocket, with the curcumin molecule making key contacts with Gly20, Gly21, Gly109, Thr132, and Asn165 and residues at the sites of Gly21, Gly22, Gly72, Thr133, and Asn166 in *B. subtilis* FtsZ (Figure 4) [66]. More recently, Morão et al., showed that a molecular simplified version of curcumin where its β-diketone moiety had been substituted with a monocarbonyl group could disrupt the divisional septum of *B. subtilis* without exerting a direct inhibition of FtsZ. These findings suggest that the simplified curcumin exerts its antibacterial action largely through membrane permeabilization, with disruption of the membrane potential necessary for FtsZ intra-cellular localization [23].

### 4.4. Induction of Oxidative Stress and Programmed Cell Death 

Traditionally, programmed cell death (PCD) is an important biological and pathological process in the life-cycle of eukaryotic multicellular organisms [67]. Similarly, monocellular organism such as bacteria can activate signaling pathways, leading to cell death within a colony. In bacteria, many factors, including stress response, developmental phase, genetic transformation, and biofilm formation contribute to the induction of bacterial programmed apoptotic-like death processes [67]. The physiological and biochemical hallmarks of apoptotic-like death in terminally stressed *E. coli* involve the production of reactive oxygen species (ROS), chromosomal condensation, extracellular exposure of phosphatidylserine, DNA fragmentation, membrane potential (ΔΨ) dissipation, and loss of structural integrity, all markers of eukaryotic apoptosis [68]. 

**Figure 5:**
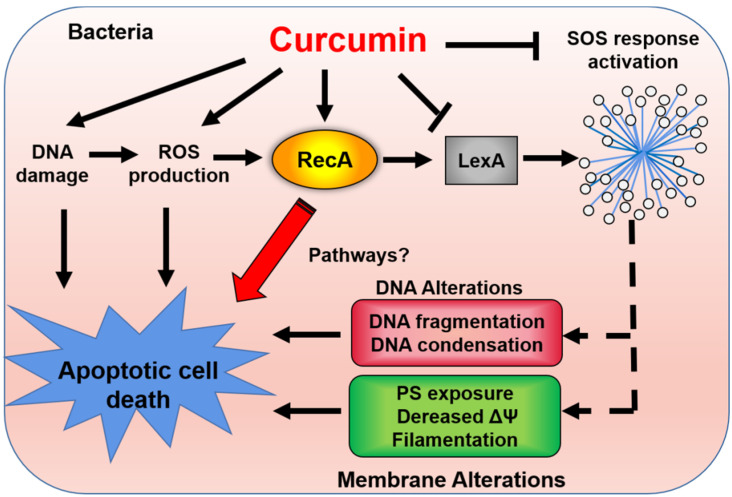
Curcumin induces oxidative stress, DNA damage, and apoptotic-like death in bacterial cells. Stress caused by drugs or other factors in bacterial cells could induce the production of ROS or DNA damage, following by activating the expression of RecA, DNA fragmentation, decreased membrane potential (ΔΨ), and formation of filamentation, finally leading to cell death. In response to this stress, the SOS response network is usually activated and plays a protective role in bacterial survival [68].

ROS-mediated cell death results from the damaging effects of the superoxide anions (O_2_^•−^), hydrogen peroxide (H_2_O_2_), and hydroxyl radicals (OH•) on bacterial cellular components (DNA, membrane lipids, and proteins) [69]. Curcumin at MIC concentrations induces the production of ROS in bacterial cells, resulting in an apoptosis-like response in *E. coli*, including the accumulation of ROS, membrane depolarization, and increase of Ca^2+^ influx [50]. At the genetic level, curcumin induced the upregulation of RecA protein expression, which mediates apoptotic-like death processes in bacteria [50]. In line with this finding, *E. coli* RecA knock-outs displayed curcumin resistance, consolidating the conclusion that curcumin-induced cell death in *E. coli* is dependent on apoptotic pathways [50]. In addition, curcumin has been shown to downregulate the expression of genes that mediate the SOS response in bacteria, which rescues the cell from DNA damage and is involved in biofilm formation and division [68]. LexA is a DNA-binding transcriptional repressor that regulates genes involved in the SOS response [70]. Recent studies indicated that curcumin inhibited the SOS responses caused by UV-induced DNA damage in *Salmonella typhimurium* and *E. coli* by suppressing the expression of LexA. The inhibitory effects of curcumin on biofilm formation and cell division mentioned above are likely associated with its inhibitory effects on the bacterial SOS response. Curcumin has also been shown to directly interact with bacterial DNA to produce a bacteriostatic effect [50]. We have provided an overview of curcumin-induced bacterial cell death in Figure 5. 

### 4.5. Phototoxicity

Curcumin absorbs blue light in the range of 455–460 nm and can be employed as an effective photosensitizer to promote the success of photodynamic processing [19]. This photosensitizing property has been exploited to induce phototoxicity in Gram-positive and -negative bacterial cells under blue light irradiation [50,71]. It is noteworthy to mention that Gram-positive bacteria are known to be more sensitive and are easily killed by photosensitizers compared to Gram-negative bacteria [50]. This difference may be related to the more robust outer membrane structure of Gram-negative bacteria compared to the more porous cytoplasmic membrane structure of Gram-positive cells in which allowed photosensitizers more easily penetrate into cells [72]. Recently, it was found that ethylene diamine tetraacetic acid (EDTA), which permeabilizes the cell membrane, could significantly enhance the antibacterial effect of blue light-activated curcumin in *S. aureus* and *S. mutans* cells [73]. 

In the past 10 years, researchers have developed a working understanding of the molecular mechanisms of curcumin-induced phototoxicity, although the precise molecular mechanism is still unclear [19]. It has been demonstrated that the antibacterial effect of blue light-activated curcumin involves an autoxidation to generate ROS, which in turn damage lipids, protein, and DNA, finally leading to bacterial cell death [50]. Jiang et al., showed that blue light-activated curcumin could significantly increase the levels of intracellular ROS and membrane damage in *S. aureus* [74]. A recent study showed that curcumin-mediated phototoxicity involves the direct induction of DNA damage and protein degradation, eradication of biofilms and inhibition of virulence genes (e.g., inlA, hlyA, and plcA) in *Listeria monocytogenes* [75]. Chen et al., showed that the process of curcumin-mediated phototoxicity is temperature dependent [76]. Very recently, it was reported that curcumin could be employed as a coating on the surface of the endotracheal tube, (which was considered the primary cause of ventilator-associated pneumonia), capable of a robust photodynamic inactivation under blue light activation (at 450 nm) against *E. coli*, *S. aureus*, and *P. aeruginosa* [77]. This photodynamic activity of curcumin provided a novel application in avoiding ventilator-associated pneumonia in patients.

### 4.6. Curcumin Perturbs Bacterial Cell Metabolism 

Many antibiotics, such as β-lactams, aminoglycosides, and quinolones, have been widely used in clinical practice, and the primary mechanisms of action have been well-established [1]. However, more recent metabolomics studies from high-throughput technologies have indicated that, in addition to these distinct mechanisms, subsequent metabolic changes that occur downstream of the interaction of the antibiotics with their primary targets also play an important role in their antibacterial-killing mechanism [78]. It has been reported that L-serine supplementation could sensitize *E. coli* to gentamicin by promoting the production of NADH and ROS production, which also mediated the bacterial killing of curcumin [79]. Adeyemi et al., reported that curcumin treatment of *S. aureus* impacts the levels of kynurenine, nitric oxide, and total thiol levels, indicating that perturbations in the aforementioned metabiotic pathways contribute to the antibacterial killing mechanism of curcumin [80]. The activation of the kynurenine pathway likely produces a decrease in the cellular L-tryptophan pool available to support bacterial growth, thereby starving bacterial cells of an essential nutrient [80]. 

### 4.7. Curcumin Regulates Intracellular Bacterial Proliferation

Curcumin is a powerful immune-regulator, with a proven ability to modulate host defenses against intracellular bacterial infections [81]. Marathe et al., showed that pre-treatment of macrophages with curcumin attenuated *Listeria monocytogenes* and *Shigella flexneri* intracellular infection, albeit the pre-treatment had the opposite effect on infection by *Salmonella enterica* serovar Typhimurium, *S. aureus*, and *Yersinia enterocolitica*, which were aggravated by curcumin [81]. This differential effect may be attributed to the membrane-stabilizing effect of curcumin wherein *S. enterica* serovar Typhimurium, *S. aureus*, and *Y. enterocolitica* have acquired machinery that inhibits the fusion of the pathogen-containing vacuole with lysosomes [82]. By contrast, *Listeria monocytogenes* and *S. flexneri* in the host cells can escape into the cytosol and prevent lysosomal degradation [83]. Recent studies also indicated that curcumin can protect human macrophages against *Mycobacterium tuberculosis* infection by inducing apoptosis, autophagy, and the activation of nuclear factor-kappa B (NF-κB) [84]. To date, the key targets of curcumin in the host that governs the growth and proliferation of intracellular pathogens are still unclear, and the precise molecular mechanisms require further investigation.

## 5. Synergistic Antibacterial Effects of Curcumin with Antibacterial or Non-Antibacterial Agents 

Synergistic antibacterial effects between antibiotics are strictly defined microbiological phenomena, requiring two bioactive agents to exhibit a greater effect in bacterial killing than the added effects of each constituent [85]. 

Several studies have shown that curcumin exhibits synergistic antibacterial effects when combined with traditional antibacterial agents (e.g., polymyxins, meropenem, oxacillin, tetracycline, ciprofloxacin, ampicillin, norfloxacin), natural products (e.g., epigallocatechin gallate, berberine) or metals (e.g., Cu^2+^, Zn^2+^, and Fe^3+^) [86,87,88,89]. In the proceeding discussion, we summarized these potential combinations and discussed their various mechanisms of action.

### 5.1. Synergistic Effect between Curcumin and Antibacterial Agents

#### 5.1.1. Curcumin and Polypeptide Antibacterial Drugs

In the clinic, vancomycin and polymyxins (including polymyxin B and E, also called colistin) are commonly employed as antibacterial drugs against MDR Gram-negative and Gram-positive bacteria, respectively [90]. The emergence of polymyxin- and vancomycin-resistant bacteria has posed a huge challenge and medical burden. 

The well-accepted primary mechanism of action of polymyxins is through spatially displacing the cations (e.g., Ca^2+^ and Mg^2+^) in the Gram-negative outer membrane and binding to the lipid A component of the lipopolysaccharide (LPS), subsequently disrupting the stability of both the outer and inner membranes, ultimately leading to bacterial cell lysis [91]. Recent studies also indicated that polymyxins can also induce the production of excessive ROS (i.e., OH^•^) in bacterial cells, leading to oxidative stress-dependent cell death [92]. Polymyxin B in combination with curcumin showed a marked synergetic effect against polymyxin-susceptible and -resistant Gram-positive (e.g., *Enterococcus, S. aureus,* and *Streptococcus*) and Gram-negative (e.g., *A. baumannii*, *E. coli*, *P. aeruginosa,* and *S. maltophilia*) bacterial isolates associated isolated from traumatic wound infections [32]. This synergistic effect may be due to curcumin’s ability to permeabilize the outer membrane, which facilitates the entry of the secondary agent to enter the bacterial cells and cause cell death [24]. In addition, this synergistic effect could be attributed to the inhibitory effect of curcumin on the activities of efflux pumps [9,24]. Curcumin and polymyxin combination treatment for bacterial infections may have another advantage, i.e., significant improvement in the therapeutic index of polymyxins by additionally inhibiting polymyxin-induced cytotoxicity, neurotoxicity, and nephrotoxicity, which is beyond antibacterial activity [93]. This combination may have a powerful application in clinical practice and warrants clinical trials.

Vancomycin is a glycopeptide antibiotic that inhibits a specific step in the synthesis of the peptidoglycan layer in Gram-positive bacteria. It has been reported that curcumin combined with vancomycin showed a synergistic effect against MDR clinical *K. pneumoniae* isolates [94]. This potential mechanism may be dependent on the synergistic effect of cell membrane permeability [94]. Moreover, curcumin could also attenuate vancomycin-induced nephrotoxicity by inhibiting oxidative stress and the inflammation response in a rat model [94]. 

#### 5.1.2. Curcumin and β- Lactam Antibacterial Drugs 

β-lactam antibiotics are the most widely used antibacterial agents worldwide. β-lactamases confer significant antibiotic resistance to their bacterial hosts by hydrolyzing the amide bond of the four-membered β-lactam ring of β-lactam antibiotics, which include four classes of drugs, i.e., penams (penicillins), cephems (cephalosporins), monobactams, and carbapenems [95]. It has reported that a curcumin and meropenem combination displayed markedly synergistic or additive effects against antibiotic-susceptible and -resistant Gram-positive (*E. faecalis*) and carbapenem-associated MDR *A. baumannii*, *P. aeruginosa*, and *K. pneumoniae* isolates via the observation of MICs [86]. A report by Yadav et al., showed that a water-soluble curcumin derivative could reverse meropenem resistance by targeting the activity of carbapenemases and the AcrAB-TolC multidrug efflux pump system [96]. Mun et al. showed that a curcumin combination with oxacillin and ampicillin exhibited a marked synergistic effect against *S. aureus* ATCC (American Type Culture Collection) 25,923 (methicillin-sensitive strain) [97]. Similarly, in another study, BDMC in combination with oxacillin showed a marked synergistic effect against *S. aureus* ATCC 33,591 (methicillin-resistant strain) and clinical MRSA isolates [98]. The potential mechanism may be dependent on the expression of the mecA gene that encodes penicillin-binding protein 2a (PBP2a), which governs the resistance of MRSA isolates to β-lactam antibiotics [98]. Sasidharan et al. found that curcumin in combination with third-generation cephalosporins (e.g., cefaclor, cefodizime, and cefotaxime) showed marked synergistic effect against *S. aureus*, *B. subtilis,* and *E. coli*, which are also associated with infectious diarrhea [87]. There was no increased toxic effects between these combinations [87]. These results indicated curcumin and cephalosporin combination are promising therapeutic options for infectious diarrhea disease.

#### 5.1.3. Curcumin and Aminoglycoside Antibacterial Drugs

Aminoglycosides are potent, broad-spectrum antibiotics that act through inhibition of protein synthesis by irreversibly binding to 30S ribosomal subunits [99]. A report by Teow et al., stated that curcumin in combination with two aminoglycoside antibiotics (e.g., amikacin and gentamicin) showed a powerful synergistic effect against *S. aureus* strains, and these synergistic effects were stronger than that of curcumin in combination with ciprofloxacin [100]. Notably, this difference in synergistic effect may be related to the difference in the primary targets between quinolone and aminoglycosides against bacteria [101]. The potential action mechanism is related to the inhibition of biofilm formation, which was evident by the significant inhibition of their combination of the swarming motilities and the mRNA expression of several key QS regulatory genes (e.g., lasI, lasR, rhlI, and rhlR) [100]. In addition, it has been reported that curcumin can also attenuate gentamicin-induced nephrotoxicity and neurotoxicity by inhibiting oxidative stress and cell apoptosis in a rat model [102]. Therefore, the combination between curcumin and aminoglycosides can not only improve the antibacterial effectiveness but can also decrease the toxic effects of gentamicin.

#### 5.1.4. Curcumin and Macrolide Antibacterial Drugs

Azithromycin is a macrolide antibiotic, which can exhibit a good antibacterial effect by inhibiting bacterial protein synthesis, quorum-sensing, and the formation of biofilms. In clinical practice, azithromycin has been used in treating respiratory, urogenital, dermal, and other bacterial infections [103]. Bahari et al., found that curcumin in combination with azithromycin showed a synergistic effect against *P. aeruginosa* PAO1, and the value of FICI was 0.25 [100]. The potential action mechanism may be similar to the above-mentioned combination of curcumin and gentamicin [100]. Erythromycin is in a class of medications called macrolide antibiotics. The action mechanism involves the blockade of bacterial growth. In a rat model, oral administration of curcumin (50 mg/kg) and erythromycin (20 mg/kg) significantly inhibited the growth of MRSA isolates in bone tissue compared to either administered alone [11]. The curcumin and erythromycin combination also significantly alleviated bone infection and the inflammatory response [11].

#### 5.1.5. Curcumin and Quinolone Antibacterial Drugs

There was a marked synergistic effect in curcumin combination with two quinolone antibiotics (e.g., ciprofloxacin and norfloxacin) against the *S. aureus* ATCC 33,591 strain and clinical MRSA isolates [97]. On the contrary, curcumin treatment reduced the antimicrobial activity of ciprofloxacin against *Salmonella typhimurium* and *Salmonella typhi* [97]. This may be related to the antioxidant property of curcumin and its inhibition of the expression of interferon γ (IFNγ) in vitro and in a mouse model [97]. 

### 5.2. Curcumin and Natural Products

#### 5.2.1. Curcumin and Berberine 

Berberine is a benzylisoquinoline alkaloid compound and has antimicrobial properties against both Gram-negative and Gram-positive bacteria [104]. Berberine has been widely used in traditional Chinese and native American medicines. FtsZ protein is an important target of berberine in inhibiting bacterial division [105]. Interesting, co-encapsulation of berberine and curcumin in liposomes decreased their MICs against MRSA by 87% and 96%, respectively, as compared to their free forms, with an FICI of 0.13, indicating a synergistic effect [88]. However, the synergistic effect in their combination in native form was not detected. In addition, co-treatment of berberine and curcumin in liposomes also significantly improved intracellular infection and the inflammation response in macrophages following MRSA infection. Mechanically, the synergistic effect between curcumin and berberine is partly dependent on the inhibition of biofilm formation and improvement of their solubilities [88]. Additionally, similar to curcumin, berberine is also an FtsZ inhibitor and inhibits bacterial cell division [104]. Therefore, this synergistic effect between curcumin and berberine may also be partly dependent on the inhibition of FtsZ assembly. 

#### 5.2.2. Curcumin and Epigallocatechin Gallate

Epigallocatechin-3-gallate (EGCG) is a polyphenol found in green tea, which, similar to curcumin, has been linked with health benefits and has significant antimicrobial activity against some MDR pathogens, including MDR *S. maltophilia*, *A. baumannii,* and *S. aureus* [106]. In vitro, it has been found that curcumin in combination with EGCG exhibited a marked synergistic effect against MDR *A. baumannii* [107]. A possible explanation for the synergy between curcumin and EGCG could be disruption of the outer membrane and facilitation of curcumin to enter bacterial cells [108]. In another study, it was suggested that inhibition of acylhomoserine lactone-mediated biofilm formation may contribute to this synergistic effect, and investigations of precise mechanisms are still required [109].

### 5.3. Curcumin and Metals 

Many metals have been used as antimicrobial agents due to the antiquity and potential molecular mechanism involved in oxidative stress, protein dysfunction or membrane damage in bacterial cells [110]. A copper (II) sulfate pentahydrate–curcumin complex (Cu-CUR), iron (III) nitrate nonahydrate–curcumin complex (Fe-CUR), and zinc (II) chloride–curcumin complex (Zn-CUR) all significantly inhibited cell growth in *P. aeruginosa* PAO1 compared to curcumin treatment alone [111,112]. Furthermore, the authors found that the Cu–CUR complex significantly inhibited the formation of the biofilm and the production of QS-related virulence factors of *P. aeruginosa* PAO1 [89]. Consistently, the synergistic activity of curcumin and silver/copper nanoparticles (NPs) was detected against the cell growth and biofilm formation of *S. aureus* and *P. aeruginosa* compared to curcumin, AgNPs or CuNPs alone [113]. These marked synergistic effects may be related to the improvement of curcumin or intracellular uptake of curcumin [114]. 

## 6. Safety of Curcumin

Curcumin has been proven to be safe and tolerable across various animal studies as well as clinical trials [115,116,117]. Orally administered curcumin at the dose of 50, 250, 480, and 1300 mg/kg body weight for 13 weeks did not exhibit acute toxicity in rats [118]. However, some abnormal effects including increased liver weight, stained fur, discolored faces, and hyperplasia of mucosal epithelium in the cecum and colon were observed in animals from the highest dosage group (2600 mg/kg body weight). Orally administered curcumin at 100, 200 or 400 mg/kg/day has been shown to effectively inhibit acute liver damage, nephrotoxicity, and nerve damage caused by colistin, aflatoxin B1, carbon tetrachloride, and cadmium [21,119,120,121,122] in rat or mouse models. In an infection model, oral administration of curcumin at 25 or 50 mg/kg body weight for two weeks could significantly ameliorate the *H. pylori* infection-induced inflammation response in gastric tissues of mice [123]. A phase I human trial showed that oral administration of curcumin in some cancer patients at a dose of 8 g/day for three months did not show any adverse effects, albeit some adverse effects were detected when the patients were administered a higher dose of 12 g/day [124]. The results of a 4-month phase I clinical trial in cancer patients showed that oral curcumin at a dose of 3.6 g/day significantly inhibited levels of serum prostaglandin E 2 (PGE2) production, a biomarker of the inflammatory response. Notably, no adverse effects were reported in the curcumin treatment cohort [125]. Consistently, a triple blinded clinical trial showed that a combination of 500 mg curcumin (equal to 8.33 mg/kg body weight) and 40 mg famotidine daily for one month significantly decreased the rate of *H*. *pylori* infection in patients [126]. Collectively, these studies indicated that the therapeutic dose of curcumin is far lower than the dosages at which toxicity is observed, thus giving curcumin a good therapeutic index.

## 7. Nano-Formulations of Curcumin

Curcumin has low water solubility (about 11 ng/mL), which results in its poor bioavailability under oral consumption [127]. Additionally, curcumin degrades rapidly, resulting in low concentrations in the blood or organs of the body, making it difficult to reach the effective concentration to treat the bacterial infection in the liver, lungs, or other organs [128]. To overcome this insufficiency of bioavailability, scientists have developed various nan-formulations of curcumin, such as lipid-based nanocarriers (e.g., liposomes, solid lipid nanoparticles, nanostructured lipid carriers, and nano-emulsion), biopolymers (e.g., nanocomposite, polymeric nanoparticles, hydrogel, and polymeric micelles), technique-based nanoparticles (e.g., spray-dried nano-formulation of curcumin, and nanofibers), and other miscellaneous types of nanocurcumin (curcumin nanocrystals, quantum dots, and graphene oxide) [18,129,130,131,132]. In addition, nanomaterial-based combinations of curcumin with other anti-bacterial agents that are effective against bacteria were also developed. Most of them are used in cancer therapy [133]. Here, we summarized the main types of nanocurcumin that are applied due to their antibacterial effect, as shown in Table 3. Their special characteristics and antibacterial activities have been well-described and addressed (see Sharifi et al.’s review paper) [132]. It is notable that there was no clinical trial to test the effectiveness of these various nano-formulations of curcumin, although they exhibited a better antibacterial effect in vitro and animal experiments by improving the solubility and biocompatibility. Therefore, more clinical trials are still required.

In addition, beyond the development of nano-formulations, other types of new formulations (e.g., inclusion technology, solid dispersion technology, microspheres, and microcapsules) were also developed to improve the solubility and bioavailability of curcumin. For example, Yaday et al. found that various cyclodextrin (CD) complexes of curcumin could enhance the solubility of curcumin > 100-fold compared with curcumin per se in water [134]. However, similar to the nano-formulations, the development of these new formulations of curcumin remains at the laboratory research stage, and there are no necessary clinical studies. 

**Table 3:** Nano-formulations of curcumin and their antibacterial effects.

Type (or Name Present in Published Literatures)	Preparation and Characterizations	Improvement in Antibacterial Activity (Accessed by MICs or Biofilm Formation)	Reference
Curcumin nanoparticles (curc-np)	Curcumin was encapsulated into a silane-hydrogel nanoparticle vehicle. Average hydrodynamic diameter at the range of 222 ± 14 nm.	In vitro, curc-np significantly inhibited the growth of MRSA and *P. aeruginosa* isolates compared to native curcumin. In a mouse model: significantly reduced bacterial burden in MRSA-infected burn wounds compared to native curcumin administration.	[29]
Nanoparticles of curcumin (nanocurcumin)	A wet-milling technique was used to make the particle size of curcumin 2–40 nm, and nanocurcumin was freely dispersible in water.	The MICs of nanocurcumin in water were 100 μg/mL, 75 μg/mL, 250 μg/mL, 200 μg/mL, 350 μg/mL against *S. aureus*, *B. subtilis*, *E. coli*, *P. aeruginosa*, *A. niger*, much higher than native curcumin in DMSO (the corresponding MICs were 150, 100, 300, 250 and 400 μg/mL).	[28,130]
Microcapsule curcumin	Microcapsule curcumin could be prepared with gelatin and porous starch as a wall system by a spray-drying method. The size was not reported.	The MICs were 250, 250, 62.5, 125, 125, 15,7, 31.3 and 31.3 μg/mL against *E.coli, Yersinia enterocolitica*, *S. aureus*, *B. subtilis*, *B. cereus*, *A. niger*, *P. notatum,* and *S. cerevisiae*. There was no comparation with native curcumin.	[135]
Sodium carboxylmethyl cellulose silver nanocomposite films-curcumin (SCMC-SNCF-CM)	SCMC-SNCF were developed from sodium carboxylmethyl cellulose (SCMC), N, N1-methylenebisacrylamide (MBA), and silver nitrate solution. Curcumin loading into SCMC–SNCF was achieved by a diffusion mechanism. The size was not reported.	SCMC-SNCF-CM composite showed 86% inhibition growth against *E. coli*. There was no comparation with native curcumin.	[136]
Curcumin Quantum Dots (CurQDs)	A newer two-step, bottom-up wet milling approach was used to prepare curcumin quantum dots (CurQDs), and acetone was used as a primary solvent. Average size was about 2.5 nm	The MIC of CurQDs significantly decreased to the range of 1.96–15.65 μg/mL from 175–300 μg/mL for native curcumin against all tested bacteria, including *S. aureus*, MRSA, *E. faecalis*, *K. Pneumoniae,* and *P. aeruginosa*	[137]
Poly-(lactic-co-glycolic acid) Curcumin nanocapsules(PLGA-CUR-NCs)	Curcumin (CUR) nanocapsules (NCs) were prepared by the solvent displacement method with some modifications. The detailed information has been described in a published paper. The solubility in water increased to 591–928 μg/mL, and its solubility could be regulated by changes in the oil and water ratio. The sizes were in the range of 100–1000 nm, dependent on the ratio of glucose.	The MICs of PLGA-CUR-NCs against *E. coli*, *Salmonella,* and *P. aeruginosa* decreased from 300 μg/mL to 100 μg/mL, and against *S. aureus*, *B. sonorensis,* and *B. licheniformis* decreased from 100 μg/mL to 75 μg/mL.	[138]
Nano-sized particles of curcumin	Colloids of curcumin nanoparticles with an average diameter of 20–40 nm were prepared in accordance with the method (a wet-milling technique).	Nano-curcumin could enhance the inhibition of biofilm formation in *P. aeruginosa*.	[139]
Cur/PVA/collagen composite films (CPCF)	A composite film (CPCF) containing curcumin nanoparticles, collagen, and polyvinyl alcohol (PVA). The diameter and polydispersity of the Cur/poly(ε-caprolactone)-poly (ethylene glycol)-poly(ε-caprolactone) PCEC nanoparticles were 43.63 ± 13.22 nm and 0.334 ± 0.403 nm, respectively.	There was no marked change in the MICs. The cytotoxicity of CPCF significantly decreased in human skin fibroblasts compared to native curcumin.	[140]
Curcumin-chitosan-zinc oxide (CCZ)	Curcumin and chitosan were layered on a hexagonal ZnO, and the particles were sized to about 48 ± 2 nm.	Increased antibacterial activity of the CCZ against MRSA and *E. coli* compared to native curcumin or ZnO.	[141]
Pectin/curcumin/sulfur nanoparticles films	pH-responsive pectin-based functional films were prepared by incorporating curcumin and sulfur nanoparticles (SNP). Curcumin and SNP were uniformly dispersed in the pectin to form a composite film.	The composite film exhibited enhanced inhibitory effect against *E. coli* and *L. monocytogenes*, with enhanced strong antioxidant activity.	[131]

## 8. Conclusions and Perspectives

In the past decades, the potential molecular mechanisms of curcumin’s antibacterial activities have been extensively studied, involving the disruption of the bacterial membrane, the inhibition of the production of bacterial virulence factors and bacterial biofilm formation, induction of oxidative stress leading to programmed cell death, bacterial metabolic disturbance, and phototoxicity. These characteristics also contribute to explain how curcumin acts a broad-spectrum antibacterial adjuvant, which was evidenced by the markedly additively or synergistically effect with various conventional antibiotics or non-antibiotic compounds, such as antibacterial agents, natural products, and metals. Animal experiments and human clinical trials reveal that curcumin has high safety. However, unlike curcumin as a chemotherapy drug in cancer therapy, curcumin as a potential antibacterial therapy still has many challenges: (1) the critical targets of curcumin alone or combination in bacteria and precise molecular mechanisms are poorly understood; (2) the poor solubility, low bioavailability, and rapid degradation in humans or animals when curcumin was consumed orally; (3) no effective clinical trials. In order to overcome the poor solubility of curcumin, scientists have developed various curcumin nano-formulations and they indeed exhibited better solubility and antibacterial activity compared to native curcumin. However, there is a lack of evidence-based randomized investigation especially exploring the therapeutic roles of the nanocarrier-based delivery systems in enhancing anti-bacterial actions; therefore, much needs to be explored.

## Figures and Tables

**Table 1:** Documented antibacterial activities of curcumin.

Bacteria Type	Antibacterial Activity	References
*Staphylococcus aureus*	Growth inhibition, inhibition of cell division or biofilm formation inhibition	[28,29,30]
*Staphylococcus epidermidis*	Growth inhibition or biofilm formation inhibition	[31]
*Streptococcus pyogenes*	Growth inhibition	[32]
*Bacillus subtilis*	Growth inhibition, or cell division inhibition	[23,28,30,33]
*Bacillus cereus*	Growth inhibition, or biofilm formation inhibition	[34,35]
*Listeria innocua*	Growth inhibition	[36]
*Helicobacter pylori*	Growth inhibition	[37,38,39]
*Pseudomonas aeruginosa*	Growth inhibition, biofilm formation inhibition, or inhibition of cell division	[28,29,30,33]
*Escherichia coli*	Growth inhibition, biofilm formation inhibition, or inhibition of cell division	[8,28,30,33]
*Streptococcus mutans*	Adhesion inhibition, biofilm formation inhibition	[40]
*Salmonella enterica* *serotype Typhmurium*	Growth inhibition, or inhibition of surface motility	[41,42]
*Klebsiella pneumoniae*	Growth inhibition	[8,9,33]
*Acinetobacter baumannii*	Growth inhibition, biofilm formation inhibition or inhibition of the surface motility	[8,43]
*Enterococcus faecium*	Growth inhibition	[8,28,33]
*Mycobacterium abscessus*	Growth inhibition, or biofilm formation inhibition	[44]
*Porphyromonas gingivalis*	Growth inhibition, or biofilm formation inhibition	[45]
*Clostridium difficile*	Growth inhibition	[46]

**Table 2:** Targets or action model of curcumin in the inhibition of biofilm in various bacteria.

Bacteria Type	Targets or Action Model of Curcumin	References
*Staphylococcus aureus*	By inhibiting the activity of sortase A by interaction with VAL-168, LEU-169, and GLN-172 sites based on curcumin and its analog methoxyl group on the benzene ring	[30,57]
*Enterococcus faecalis*	Unclear	[54]
*Listeria monocytogenes*	By circumventing the limitations to singlet-oxygen diffusion imposed by the extracellular matrix	[36]
*Bacillus cereus*	Unclear	[35]
*Helicobacter pylori*	By inhibiting biofilm maturation	[38]
*Pseudomonas aeruginosa*	By inhibiting the production of the QS-dependent factors, such as exopolysaccharide production, alginate production, swimming, and swarming motility of uropathogens	[30,58]
*Escherichia coli*	Similar to *Pseudomonas aeruginosa*	[58]
*Streptococcus mutans*	By inhibiting sortase A activity; suppressing the expression of genes related to extracellular polysaccharide synthesis, carbohydrate metabolism, adherence, and the two-component transduction system	[59,60,61]
*Serratia marcescens*	By inhibiting the production of violacein production in a QS-independent manner, as well as swimming and swarming motility.	[55]
*Klebsiella pneumoniae*	Unclear	[62]
*Acinetobacter baumannii*	By blocking BfmR, which is a response regulator in a two-component signal transduction system	[43]
*Aeromonas hydrophila*	Inhibition of violacein production and swimming motility	[53,63]
*Porphyromonas gingivalis*	By inhibiting the activities of Arg-- and Lys-specific proteinase (named RGP and KGP, respectively)	[45]
